# Symptoms and Cognitive Functions in Adolescents in Relation to Mobile Phone Use during Night

**DOI:** 10.1371/journal.pone.0133528

**Published:** 2015-07-29

**Authors:** Anna Schoeni, Katharina Roser, Martin Röösli

**Affiliations:** 1 Swiss Tropical and Public Health Institute, Basel, Switzerland; 2 University of Basel, Basel, Switzerland; Brock University, CANADA

## Abstract

Many adolescents tend to leave their mobile phones turned on during night, accepting that they may be awakened by an incoming text message or call. Using self-reported and objective operator recorded mobile phone use data, we thus aimed to analyze how being awakened during night by mobile phone affects adolescents’ perceived health and cognitive functions. In this cross-sectional study, 439 adolescents completed questionnaires about their mobile phone use during night, health related quality of life and possible confounding factors. Standardized computerized cognitive tests were performed to assess memory and concentration capacity. Objective operator recorded mobile phone use data was further collected for 233 study participants. Data were analyzed by multivariable regression models adjusted for relevant confounders including amount of mobile phone use. For adolescents reporting to be awakened by a mobile phone during night at least once a month the odds ratio for daytime tiredness and rapid exhaustibility were 1.86 (95% CI: 1.02–3.39) and 2.28 (95% CI: 0.97–5.34), respectively. Similar results were found when analyzing objective operator recorded mobile phone use data (tiredness: 1.63, 95% CI: 0.94–2.82 and rapid exhaustibility: 2.32, 95% CI: 1.01–5.36). The cognitive tests on memory and concentration capacity were not related to mobile phone use during night. Overall, being awakened during night by mobile phone was associated with an increase in health symptom reports such as tiredness, rapid exhaustibility, headache and physical ill-being, but not with memory and concentration capacity. Prevention strategies should focus on helping adolescents set limits for their accessibility by mobile phone, especially during night.

## Introduction

Within the last 15 years the use of mobile phones has increased remarkably in adults as well as in adolescents according to the International Telecommunication Union [1]. Many adolescents tend to leave their mobile phones turned on during night and accept that they may be awakened by an incoming text message or call. A survey conducted in 2003 in Belgium showed that 27% of 13 year olds and 43% of 16 year olds reported being disturbed in their sleep by incoming text messages, leading to an unhealthy sleep pattern [2]. A follow-up investigation one year later in the same study collective revealed substantially increased levels of tiredness for study participants who used the mobile phone more frequently during night [3]. A cross-sectional study from Munezewa et al.[4] showed that mobile phone use after lights out is associated with short sleep duration, poor sleep quality, daytime sleepiness and insomnia symptoms. Thomée et al.[5] found, in a cross-sectional analysis of data from 4,156 young Swedish adults, that being awakened by mobile phone at night was associated with current stress, sleep disturbances and symptoms of depression.

To our knowledge, no study has investigated mobile phone use during night in relation to effects on cognitive functions. However, an epidemiological study investigated if regular mobile phone use is associated with impaired cognitive functions. Abramson et al.[6] observed that mobile phone use in 317 seventh grade students from Australia was associated with faster and less accurate response on a number of cognitive tasks but speculated that these behaviours may have been learned through frequent use of a mobile phone. In a follow-up investigation one year later, in 236 of these students, changes in response times rather than in accuracy were observed, which were mainly attributed to statistical artefacts [7]. Since amount of mobile phone use in general may be related to the use during night, the observed associations on cognitive functions may be the consequence of night-time use. On the other hand, it is not clear whether the observed patterns with health outcomes are confounded by some other factors related to mobile phone use during night. A limitation of all previous studies is that they were restricted to self-reported mobile phone use data, which has been shown to be inaccurate [8–10]. Rank correlation coefficients between self-reported and objectively recorded mobile phone use varied between 0.1 and 0.9 [8–10] with a tendency for adolescents to overestimate their duration of mobile phone use but being more accurate on the frequency of mobile phone use.

In the framework of the HERMES (Health Effects Related to Mobile phonE use in adolescentS) study we aimed to evaluate how adolescents’ perceived health and cognitive functions are affected by various aspects of mobile phone use including electromagnetic field exposure. In this paper, we focus on the question whether being awakened during night by an incoming text message or call is associated with negative consequences by using both self-reported mobile phone use data and objective operator recorded mobile phone use data.

## Methods

### Ethics Statement

Ethical approval for the conduct of the study was received from the ethical committee of Lucerne, Switzerland (Dienststelle Gesundheit, Ethikkommission des Kantons Luzern, Schweiz) on May 9^th^, 2012 (Ref. Nr. EK: 12025). The ethical approval was based on the information sheet of the study, the study protocol and summary and questionnaires for the involved parents and adolescents. Written informed consent was obtained from the adolescents and their parents for the participation in the study and for providing the mobile phone operator data.

### Study population

439 students (participation rate: 36.8%) aged 12 to 17 years and attending 7^th^, 8^th^ or 9^th^ grade in 24 schools (participation rate: 19.1%) from rural and urban areas in Central Switzerland participated in the HERMES study. During a school visit between June 2012 and February 2013 the adolescents filled in a questionnaire and performed two cognitive tests using a standardized, computerized cognitive testing system. Additionally a questionnaire for the parents was distributed. The questionnaire for the parents included questions, amongst others, on the behaviour of their children, on socio-economic factors, on wireless technology at home and on child development. Parents were asked to fill out the questionnaire and send it back directly.

### Mobile phone use

The study participants were asked whether they turned off their mobile phone during night and how often they were being awakened by their own or by their roommate’s mobile phone. Among those who reported being awakened by mobile phone, they were asked whether they text or call back (referred to as being responsive) during night. For the analysis with the self-reported mobile phone use data, four categories were created. The reference category included those 27 study participants not owning a mobile phone and those reporting to turn off their mobile phone during night (“Mobile phone turned off / no mobile phone”). The other categories referred to those who reported not turning off their mobile phones. The second category included those not awakened by mobile phone during night (“Not being awakened”); the third category included those who reported being awakened by mobile phone at least once a month (“Being awakened (≥1x per month)”); and the forth category is a subgroup of those being awakened who additionally reported to be responsive when being awakened during night (“Being awakened and responsive”).

Informed consent to obtain objective mobile phone use data from the mobile phone operators was given by 233 out of 439 study participants and their parents. Data were obtained for up to 6 months before date of investigation. For each participant the number of nights with incoming calls and text messages were calculated by defining night-time use from 11pm to 6am on week days and 12midnight to 8am for Friday and Saturday nights. The mobile phone operators record the time of an incoming text message or call only when the mobile phone is turned on. If the mobile phone is turned off during night, the time of an incoming text message is recorded as soon as the mobile phone is turned on. Thus, when text messages or calls were recorded during night, the mobile phone was turned on.

For the analysis with the objective operator recorded mobile phone use data two categories were created. The 27 study participants not owning a mobile phone were added to the reference group together with those having incoming calls and text messages less than once per month (“No mobile phone / not being awakened (<1x per month)”). An additional analysis was done, omitting study participants not owning a mobile phone.

### Health outcomes

In the written questionnaire headache was assessed using the six-item Headache Impact Test [11]. A summary score of all six items can range from 36 to 78. A summary score of 49 or less is considered as “headache has no impact on your life,” 50 to 55 is considered as “headache has some impact on your life,” 56 to 59 as “headache has substantial impact on your life” and 60 or more as “headache has a very severe impact on your life.” A binary variable was created by using 56 as the cut-off value. Tiredness, lack of energy, lack of concentration and rapid exhaustibility (referred to as exhaustibility) were assessed using a four-point Likert scale with categories “never,” “rare,” “moderate” and “severe.” Binary variables were created by combining answer categories “never” with “rare” and “moderate” with “severe”. Physical well-being was assessed using the dimension “Physical Well-being” from the Kidscreen-52 questionnaire. This dimension includes five questions exploring the level of adolescent’s physical activity, energy and fitness [12,13]. A binary variable was created by using the mean minus half a standard deviation as the cut-off, which is suggested as the guiding principle according to the official Kidscreen questionnaire handbook.

### Cognitive tests

Cognitive functions were assessed with a standardized, computerized cognitive test battery (FAKT-II, *Frankfurter Adaptiver Konzentrationsleistungs-Test-II* [14] and a subtest of the IST, *Intelligenz-Struktur-Test 2000R* [15]). Concentration capacity which includes the power of concentration, the accuracy of concentration and the homogeneity of concentration was measured with the FAKT-II. By means of discrimination tasks, the study participant has to discriminate as accurately and as quickly as possible between target and non-target items by pressing “0” for non-target items and “1” for target items. Items with either two or three points in either a circle or a square appeared. Target items have either two points in a square or three points in a circle. Other combinations act as non-target items. Before starting the 6-minute test, the study participants performed a trial-run.

Power of concentration is a measure of the working rate. It measures the number of displayed items per 10 seconds. The higher the power of concentration, the faster the study participant worked. Accuracy of concentration is a measure of the relative correctness. It measures the percentage of non-false items that have been processed. The higher the accuracy of concentration, the more precise the study participant worked. Homogeneity of concentration is a measure of the uniformity of the working rate. It measures the variance of the time an item is displayed. The higher the homogeneity of concentration, the more uniform the study participant worked. These three measures were used for statistical analyses.

Verbal and figural memory was measured with the subtest of the IST. In the verbal memory task, word groups have to be memorized in one minute time. After one minute the study participants give an account of the word groups that have been memorized. In total 10 points can be achieved by remembering the correct word groups. In the figural memory task, pairwise symbols have to be memorized in one minute time. After one minute, one part of the pairwise symbol is shown and the matching part has to be found. A total of 13 points can be achieved. For both the verbal and figural tests, 2 minutes are given to complete the test. For the “overall memory” score, the figural and verbal memory scores are summed. Therefore a total of 23 points can be achieved. For the statistical analyses of verbal and figural memory as well as memory overall the continuous test score values were used as outcome. The cognitive tests, conducted during school time, were administered by two study managers.

### Covariates

The written questionnaires for the study participants included questions about age, sex, class level, nationality, school level, physical activity, alcohol consumption and frequency of mobile phone calls. The questionnaires for the parents included questions, among others, on socio-economic factors.

### Statistical Analysis

The association between mobile phone use during night and symptoms was analyzed by logistic regression and risk estimates are expressed as odds ratios. The association with cognitive functions was analyzed with linear regression models and thus model coefficients refer to the increase in test score.

**Fig 1:**
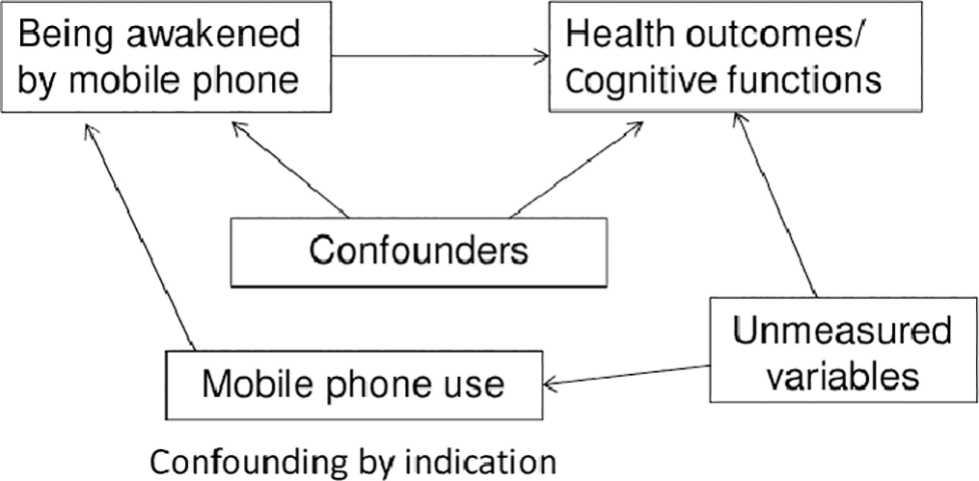
Confounding by indication.

A first model (adjusted 1) was adjusted for age, sex, class level (7^th^, 8^th^ or 9^th^ grade), nationality, school level (college preparatory high school or high school), physical activity, alcohol consumption and education of parents. Since total amount of mobile phone use is associated with night-time use, a second model (adjusted 2) was calculated with additional adjustment; we used self-reported frequency of mobile phone calls per day in the analysis with self-reported data, and recorded duration of mobile phone calls per day in the analysis with operator recorded data. This model addresses potential confounding by indication, which refers to (unmeasured) variables related to mobile phone use and to our outcomes as depicted in Fig 1. The effect of being awakened by mobile phone on the risk of being physically ill/ impaired cognitive functions will be confounded if being awakened by mobile phone is more likely in individuals with higher mobile phone use. Mobile phone use is a risk factor for our outcomes because mobile phone use has a direct causal effect on our outcomes, since both mobile phone use and our outcomes are caused by unmeasured variables (e.g. personality). We suspect confounding by indication because numerous studies observed cross-sectional associations between amount of mobile phone use and symptoms such as fatigue [16–18], depressed mood [5], and headache [17,19]. We hypothesize that such associations may be, at least partly, not directly caused by mobile phone use itself but by unmeasured factors related to mobile phone use such as personality. In epidemiological terms this means that there is a backdoor path between the exposure and the outcomes through the unmeasured variables. This backdoor path could be eliminated by conditioning (adjusting) on the unmeasured variables. Because one cannot adjust for these unmeasured variables, the backdoor path can also be blocked by conditioning (adjusting) on mobile phone use [20]. The results from the analyses with mobile phone adjustment (adjusted 2) thus represent the effect mediated by unmeasured variables (e.g. sleep disturbances) due to nocturnal mobile phone use, whereas for the results of the adjusted 1 model other factors related to mobile phone use in general may also play a role.

Linear regression imputation (14 missing values for alcohol consumption) or imputation of a common category (77 missing values for educational level of the parents) was used to impute missing values in the confounder variables. Statistical analyses were carried out using STATA version 12.1 (StataCorp, College Station, USA).

## Results

In total, 439 study participants took part at the baseline investigation. Of those, objective operator data for 233 study participants was obtained. Table 1 shows the distribution of key socio-demographic characteristics in the whole sample (N = 439) and in the subgroup of study participants for which we obtained objective operator recorded data (N = 233).

**Table 1:** Distribution over socio-demographic characteristics from the whole sample (N = 439) and from the subgroup of study participants with operator recorded data (N = 233).

	N = 439	Prop(%)	N = 233	Prop(%)
*Age (years)*				
12–13	44	10.0	28	12.0
>13–14	200	45.6	105	45.1
>14–15	142	32.3	80	34.3
>15	53	12.1	20	8.6
*Sex*				
Female	265	60.4	150	64.4
Male	174	39.6	83	35.6
*Class level*				
7th grade	105	23.9	52	22.3
8th grade	293	66.8	172	73.8
9th grade	41	9.3	9	3.9
*School level*				
College preparatory high school	99	22.5	66	28.3
High School	340	77.5	167	71.7
*Nationality*				
Swiss	348	79.3	189	81.1
Swiss and other	62	14.1	31	13.3
Other	29	6.6	13	5.6
*Physically active*				
Yes	379	86.3	202	86.7
*Number of days with alcohol consumption*				
None	304	69.2	156	67.0
One or less than one per month	99	22.6	55	23.6
More than one per month	36	8.2	22	9.4
*Highest education of parents*				
No education	3	0.7	-	-
Mandatory school / High school	9	2.0	4	1.7
Training school	233	53.1	118	50.6
College preparatory high school	29	6.6	14	6.0
College of higher education	130	29.6	78	33.5
University	35	8.0	19	8.2
*Frequency [x/d] of mobile phone calls (self-reported; N = 439)*				
never; 0 x/d	27	6.1	-	-
>0 to ≤0.5 x/d	216	49.2	-	-
>0.5 to ≤1 x/d	71	16.2	-	-
>1 to ≤5 x/d	115	26.2	-	-
>5 x/d	10	2.3	-	-
*Duration [min/d] of operator recorded mobile phone calls (N = 233)*				
≤1 min/day	-	-	151	64.8
>1 to ≤2 min/day	-	-	36	15.4
>2 to ≤5 min/day	-	-	26	11.2
>5 to ≤10 min/day	-	-	12	5.2
>10 min/day	-	-	8	3.4

In total 412 (93.9%) study participants owned a mobile phone. Median age was 13.9 years (range 12–17 years). One study participant did not answer questions about nocturnal mobile phone use and was therefore excluded from analyses with self-reported data. Objective operator recorded mobile phone use data were obtained from this particular study participant and therefore this participant was included in the analyses with operator recorded mobile phone use data.

From 438 study participants, 126 (28.7%) either had no mobile phone or indicated to switch off their mobile phone during night and reported not being awakened by any other mobile phone. Of study participants who did not switch off their mobile phone at night 216 (49.3%) indicated not being awakened by a mobile phone, while 96 (21.9%) indicated being awakened at night by a mobile phone at least once a month. Of the 96 study participants who are awakened at night by a mobile phone, 61 (67.8%) reported to respond to incoming text messages or calls during night. Of the 233 study participants from which operator recorded data were obtained 110 (42.3%) received an incoming text message or call during night at least once a month.

The Spearman correlation of self-reported frequency of being awakened and the corresponding operator data derived frequency was 0.30. Self-reported frequency of being awakened was also correlated with self-reported frequency of mobile phone calls per day: 0.32. Operator recorded frequency of being awakened was correlated with objective recorded mobile phone use: 0.52. Spearman correlation of self-reported call duration and operator recorded call duration per day was 0.55. The same correlation was found for frequency of calls between self-reported and operator recorded data.

### Symptoms

Table 2 shows the association between self-reported mobile phone use during night and health symptoms. After adjusting for age, sex, class level, nationality, school level, physical activity, alcohol and education of parents (adjusted 1), increased OR for all symptoms except for lack of concentration and lack of energy were seen with significant effects for: tiredness (OR:2.06, 95% CI:1.16–3.66), exhaustibility (OR:2.94, 95% CI:1.30–6.63), headache (OR:2.71, 95% CI:1.30–5.63) and physical ill-being (OR:2.93, 95% CI:1.54–5.57) for those reporting being awakened by mobile phone during night at least once a month. After additional adjustment for the frequency of mobile phone calls (adjusted 2) the OR decreased somewhat but the result pattern remained similar.

**Table 2:** Association between self-reported mobile phone use during night and symptoms.

	n with / without	crude	adjusted 1*	adjusted 2**
Symptom	symptoms	OR (95% CI)	OR (95% CI)	OR (95% CI)
***Tiredness*** *(N = 438)*				
Phone turned off / no phone	50/76	1	1	1
Not being awakened	96/120	1.22 (0.78–1.90)	1.23 (0.78–1.95)	1.18 (0.74–1.88)
Being awakened (≥1x per month)	56/40	**2.13 (1.24–3.65)**	**2.06 (1.16–3.66)**	**1.86 (1.02–3.39)**
Being awakened and responsive^t^	42/19	**3.36 (1.76–6.43)**	**3.33 (1.67–6.66)**	**3.04 (1.48–6.25)**
***Lack of concentration*** *(N = 438)*				
Phone turned off / no phone	22/104	1	1	1
Not being awakened	39/177	1.04 (0.59–1.85)	1.00 (0.55–1.81)	0.92 (0.50–1.69)
Being awakened (≥1x per month)	23/73	1.49 (0.77–2.87)	1.35 (0.67–2.71)	1.14 (0.55–2.38)
Being awakened and responsive^t^	16/45	1.68 (0.81–3.50)	1.57 (0.71–3.46)	1.30 (0.56–3.00)
***Exhaustibility*** *(N = 434)*				
Phone turned off / no phone	12/114	1	1	1
Not being awakened	26/188	1.31 (0.64–2.71)	1.34 (0.64–2.82)	1.18 (0.55–2.52)
Being awakened (≥1x per month)	22/72	**2.90 (1.35–6.22)**	**2.94 (1.30–6.63)**	2.28 (0.97–5.34)
Being awakened and responsive^t^	14/45	**2.96 (1.27–6.88)**	**2.79 (1.13–6.91)**	2.05 (0.79–5.33)
***Lack of energy*** *(N = 438)*				
Phone turned off / no phone	20/106	1	1	1
Not being awakened	29/187	0.82 (0.44–1.52)	0.87 (0.46–1.64)	0.78 (0.41–1.49)
Being awakened (≥1x per month)	23/73	1.67 (0.86–3.26)	1.85 (0.90–3.77)	1.45 (0.68–3.09)
Being awakened and responsive^t^	16/45	1.88 (0.90–3.97)	2.22 (0.99–4.99)	1.67 (0.71–3.96)
***Headache*** *(N = 433)*				
Phone turned off / no phone	16/110	1	1	1
Not being awakened	36/177	1.40 (0.74–2.64)	1.38 (0.71–2.66)	1.14 (0.58–2.24)
Being awakened (≥1x per month)	28/66	**2.92 (1.47–5.79)**	**2.71 (1.30–5.63)**	1.86 (0.86–4.05)
Being awakened and responsive^t^	20/39	**3.53 (1.66–7.48)**	**3.08 (1.37–6.95)**	2.00 (0.84–4.75)
***Physical ill-being*** *(N = 437)*				
Phone turned off / no phone	27/99	1	1	1
Not being awakened	78/138	**2.07 (1.25–3.44)**	**2.21 (1.29–3.79)**	**2.04 (1.18–3.53)**
Being awakened (≥1x per month)	42/53	**2.91 (1.61–5.23)**	**2.93 (1.54–5.57)**	**2.44 (1.25–4.77)**
Being awakened and responsive^t^	32/28	**4.19 (2.16–8.12)**	**4.25 (2.05–8.79)**	**3.52 (1.65–7.52)**

^t^ subgroup of the "Being awakened" group.

*adjusted for age, sex, class level, nationality, school level, physical activity, alcohol, education of parents.

**adjusted for frequency of mobile phone calls in addition to adjusted 1.

For the subgroup of people reporting to respond to a text message or call at night, OR (adjusted 1) were even larger for all symptoms with significant effects for: tiredness (OR:3.33, 95% CI:1.67–6.66), exhaustibility (OR:2.79, 95% CI:1.13–6.91), headache (OR:3.08, 95% CI:1.37–6.95) and physical ill-being (OR:4.25, 95% CI:2.05–8.79). After adjustment for the frequency of mobile phone calls (adjusted 2) OR decreased somewhat and only the OR of tiredness and physical ill-being remained significant.

**Fig 2:**
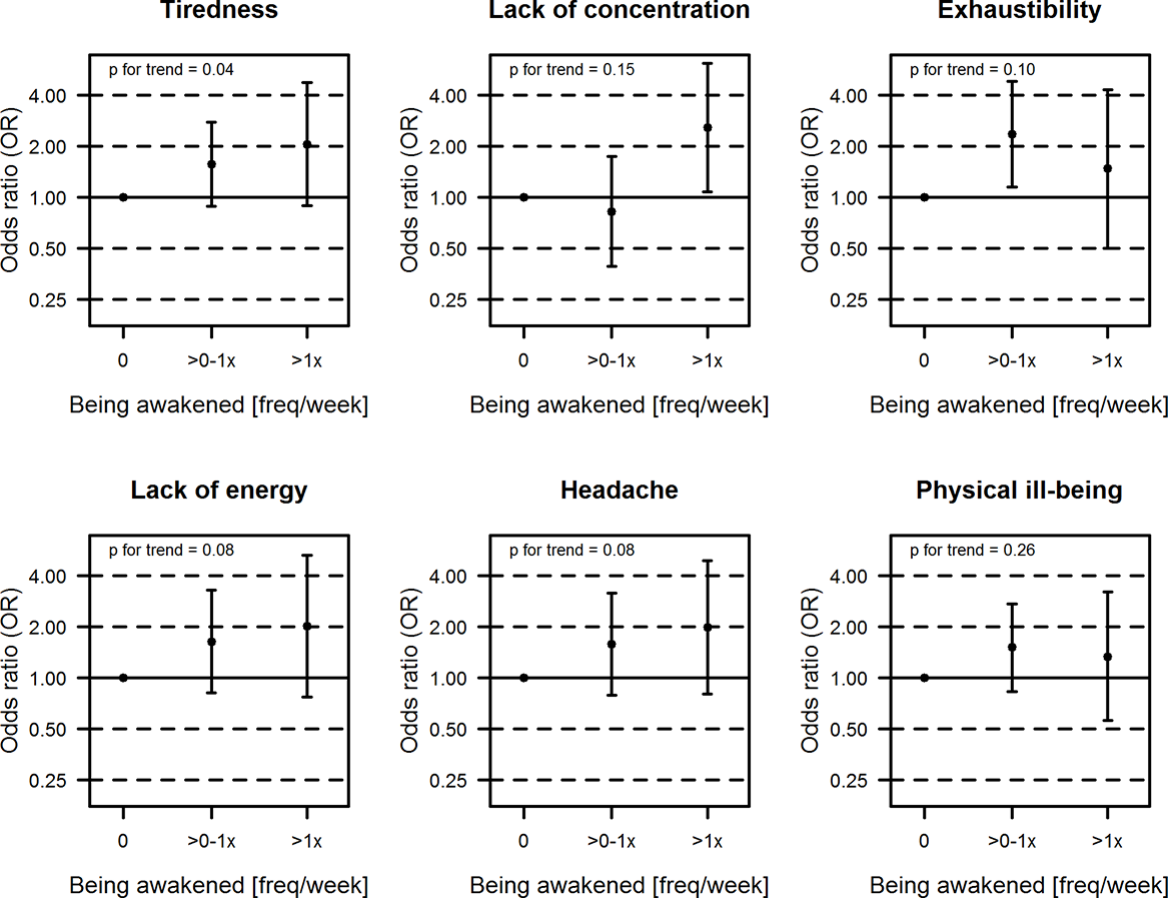
Exposure- response frequency of the association between being awakened during night and symptoms (self-reported; adjusted 2). **adjusted for age, sex, class level, nationality, school level, physical activity, alcohol, education of parents, frequency of mobile phone calls.

Fig 2 shows the exposure-response frequency of the association between self-reported mobile phone use during night and symptoms. For tiredness, lack of concentration, lack of energy and headache the OR increased with increasing number of reported awakenings per week. For exhaustibility and physical ill-being such an exposure-response pattern was not found. Tiredness showed a significant test of trend.

Table 3 shows results of the objective operator recorded mobile phone use during night. Increased OR (adjusted 1) for all symptoms were seen for participants who were awakened at least once a month by text message or call with significant results for headache (OR: 2.30, 95% CI:1.12–4.73). Additionally adjusting for mobile phone use rather resulted in an increase of the OR than in a decrease as seen for self-reported mobile phone use. In a sensitivity analysis study participants, who did not own a mobile phone, were omitted to possibly obtain a more homogenous reference group. Omitting study participants who did not own a mobile phone yielded higher OR for tiredness (OR: 1.78, 95% CI:1.01–3.14), lack of concentration (OR: 1.39, 95% CI:0.66–2.94), exhaustibility (OR: 2.72, 95% CI:1.08–6.89), lack of energy (OR: 2.18, 95% CI:0.92–5.15) and headache (OR: 3.03, 95% CI:1.33–6.91).

**Table 3:** Association between operator recorded mobile phone use during night and symptoms for the sample of 233 study participants for which operator data were obtained, together with the 27 study participants who do not own a mobile phone.

	n with /without	crude	adjusted 1*	adjusted 2**
Symptom	symptoms	OR (95% CI)	OR (95% CI)	OR (95% CI)
***Tiredness*** *(N = 260)*				
No phone / not being awakened	65/85	1	1	1
(<1x per month)				
Being awakened (≥1x per month)	60/50	1.57 (0.96–2.57)	1.53 (0.91–2.60)	1.63 (0.94–2.82)
***Lack of concentration*** *(N = 260)*				
No phone / not being awakened	25/125	1	1	1
(<1x per month)				
Being awakened (≥1x per month)	22/88	1.25 (0.66–2.36)	1.28 (0.65–2.54)	1.32 (0.65–2.67)
***Exhaustibility*** *(N = 260)*				
No phone / not being awakened	13/137	1	1	1
(<1x per month)				
Being awakened (≥1x per month)	18/92	2.06 (0.96–4.41)	2.05 (0.91–4.60)	**2.32 (1.01–5.36)**
***Lack of energy*** *(N = 260)*				
No phone / not being awakened	19/131	1	1	1
(<1x per month)				
Being awakened (≥1x per month)	17/93	1.26 (0.62–2.55)	1.28 (0.61–2.70)	1.55 (0.72–3.38)
***Headache*** *(N = 257)*				
No phone / not being awakened	21/128	1	1	1
(<1x per month)				
Being awakened (≥1x per month)	28/80	**2.13 (1.14–4.01)**	**2.30 (1.12–4.73)**	**2.17 (1.03–4.56)**
***Physical ill-being*** *(N = 259)*				
No phone / not being awakened	43/107	1	1	1
(<1x per month)				
Being awakened (≥1x per month)	42/67	1.56 (0.92–2.63)	1.55 (0.86–2.77)	1.67 (0.91–3.06)

*adjusted for age, sex, class level, nationality, school level, physical activity, alcohol, education of parents.

**adjusted for duration of mobile phone calls in addition to adjusted 1.

### Cognitive functions

Descriptive statistics for the cognitive tests are given in Table 4.

**Table 4:** Descriptive statistics for the tests of the cognitive functions.

	n^1^	mean	sd	min	median	max
Power of concentration (Number of items per 10 sec)	349	8.00	2.76	1.66	7.96	17.10
Accuracy of concentration (%)	349	79.35	5.52	67.30	78.60	98.70
Homogeneity of concentration (Variance of time)	349	25.59	16.48	4.70	22.80	100
Verbal memory (test score)	416	5.02	2.76	0	5	10
Figural memory (test score)	419	8.06	2.76	0	8	13
Memory overall (test score)	416	13.09	4.44	2	13	23

^1^ due to technical problems of the computerized testing system, data was not available for the whole sample.

**Fig 3:**
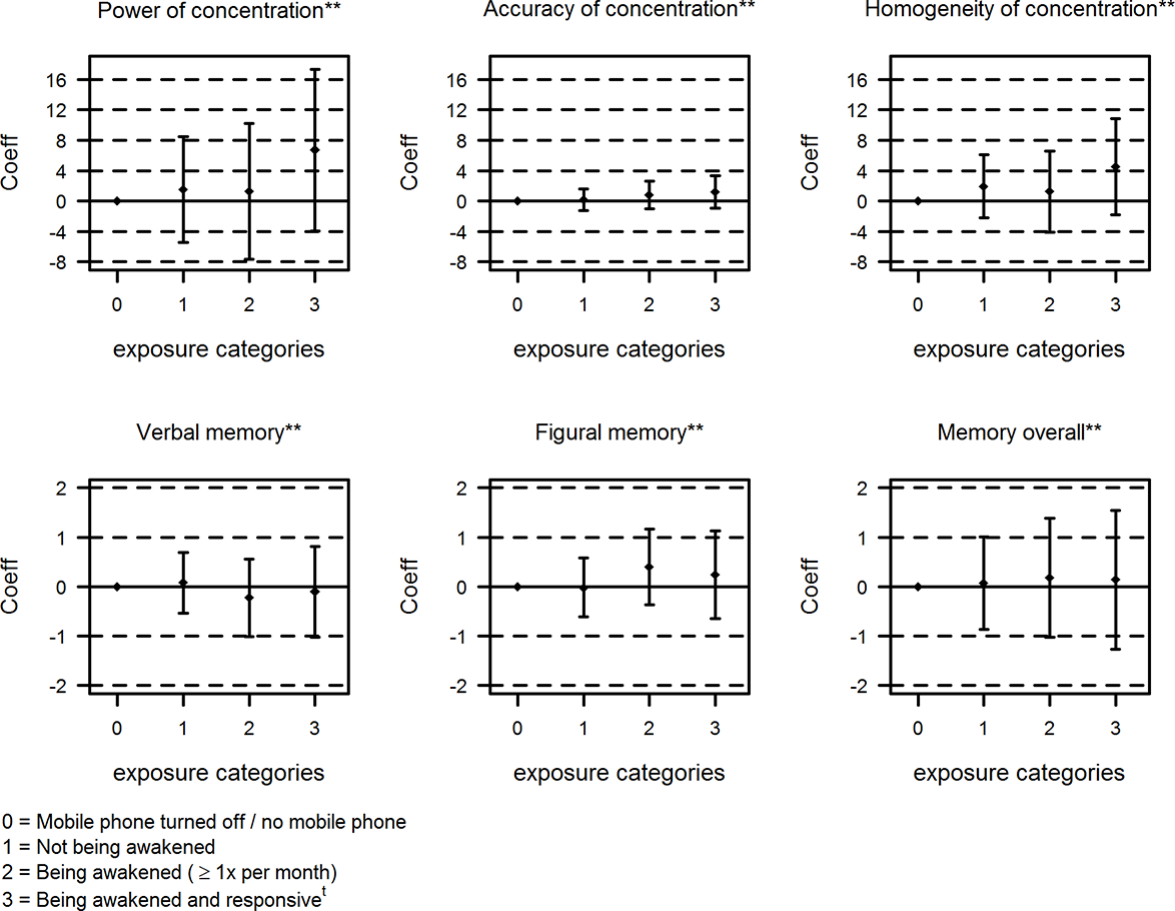
Association between self-reported mobile phone use during night and cognitive functions. ^t^ subgroup of the “Being awakened” group. **adjusted for age, sex, class level, nationality, school level, physical activity, alcohol, education of parents, frequency of mobile phone calls.

The analysis between self-reported mobile phone use during night and cognitive functions are shown in Fig 3. Power of concentration (number of displayed items per 10 seconds), as well as Accuracy of concentration (%) and Homogeneity of concentration (variance of the time an item is displayed) were not associated with self-reported mobile phone use during night.

The spearman correlations of self-reported lack of concentration and Power of concentration, Accuracy of concentration and Homogeneity of concentration measured with the cognitive tests were 0.11, 0.10 and 0.12, respectively. Furthermore no association was found when analyzing verbal, figural and overall memory with self-reported mobile phone use during night. These results were confirmed by analyzing objective operator recorded mobile phone use during night (Table 5).

**Table 5:** Association between operator recorded mobile phone use during night and cognitive functions for the sample of 233 study participants for which operator data were obtained, together with the 27 study participants who do not own a mobile phone.

		crude	adjusted 1*	adjusted 2**
Cognitive Function	n	Coeff ^1^ (95% CI)	Coeff ^1^ (95% CI)	Coeff ^1^ (95% CI)
***Power of concentration*** *(N = 210)*				
No phone / not being awakened	118	0	0	0
(<1x per month)				
Being awakened (≥1x per month)	92	0.01 (-0.75–0.76)	-0.07 (-0.81–0.68)	0.04 (-0.73–0.81)
***Accuracy of concentration*** *(N = 210)*				
No phone / not being awakened	118	0	0	0
(<1x per month)				
Being awakened (≥1x per month)	92	0.25 (-1.33–1.83)	0.24 (-1.37–1.84)	0.25 (-1.41–1.91)
***Homogeneity of concentration*** *(N = 210)*				
No phone / not being awakened	118	0	0	0
(<1x per month)				
Being awakened (≥1x per month)	92	0.11 (-4.66–4.88)	-0.62 (-5.37–4.12)	-0.14 (-5.03–4.76)
***Verbal memory*** *(N = 251)*				
No phone / not being awakened	145	0	0	0
(<1x per month)				
Being awakened (≥1x per month)	106	0.23 (-0.47–0.93)	0.16 (-0.54–0.87)	0.30 (-0.43–1.03)
***Figural memory*** *(N = 252)*				
No phone / not being awakened	145	0	0	0
(<1x per month)				
Being awakened (≥1x per month)	107	0.60 (-0.04–1.24)	0.36 (-0.24–0.97)	0.39 (-0.25–1.02)
***Memory overall*** *(N = 251)*				
No phone / not being awakened	145	0	0	0
(<1x per month)				
Being awakened (≥1x per month)	106	0.83 (-0.24–1.91)	0.54 (-0.47–1.55)	0.70 (-0.35–1.75)

*adjusted for age, sex, class level, nationality, school level, physical activity, alcohol, education of parents.

**adjusted for duration of mobile phone calls in addition to adjusted 1.

^1^ refers to change in the test score per exposure category.

## Discussion

The aim of this study was to investigate how the mobile phone use during night affects adolescents’ perceived health and cognitive functions. Our results demonstrate that mobile phone use during night is common among adolescents. Increased symptom reports were shown when adolescents are being awakened by mobile phones during night at least once a month. These findings were confirmed by analyzing objective operator recorded mobile phone use data although with wider confidence intervals due to a smaller sample size thus, except for exhaustibility and headache, not reaching statistical significance. Memory and concentration capacity were not associated with nocturnal mobile phone use.

Mobile phone use during night is likely to reduce sleep quality and sleep quantity. Several studies have shown a strong relationship between too short and poor sleep and health consequences such as fatigue [21], headache [22], subjective psychological well-being [23,24], respiratory disorders [25] or cardiovascular diseases [26,27]. The exact underlying mechanisms are not known, but may be mediated by inflammatory responses [28] or by neurophysiological mechanisms [29].

Interruption of sleep may be the underlying mechanism for the observed increase in symptoms in our study when the study participants were being awakened by mobile phone at least once a month. Even higher OR for health outcomes were found when study participants were responsive after being awakened by mobile phone. One could hypothesize that being responsive during night might cause overexcitement and thus negatively affect further sleep, leading to even less sleep compared to those only being awakened. The fact that some OR were slightly increased, even statistically significant for physical ill-being, when the study participants reported to leave their mobile phones turned on (but not report being awakened), could be due to expectation. Only the expectation of getting a call or a text message may lead to poor sleep and thus to an increase in symptoms. It can also be that some adolescents who kept the mobile phone on during night did not report being occasionally awakened by their mobile phone. However, if that would be the case, we would expect to get some significant results also for other symptoms. The exposure-response pattern of the association between self-reported mobile phone use during night and health outcomes showed that frequency in addition to being awakened also plays an important role.

Caution is needed in interpreting the directions of the associations. We hypothesize that mobile phone use during night might affect sleep which in turn might lead to more symptoms. An alternative hypothesis would be reverse causality in the sense that study participants with sleep disturbances and more health symptoms use the mobile phone during night more often than their peers who have no sleep disturbances.

Sleep plays an important role concerning health outcomes, but also in learning processes and memory consolidation [30]. Sleep contributes to memory consolidation before and after learning [31]. Furthermore, sleep is not only important for memory consolidation on long-term; it is also important on short-term [32]. Consistently reduced task-related activation in verbal short-term memory tasks were shown in sleep deprived individuals [33,34]. The same applies to concentration and attention tasks [35,36]. A characteristic of a sleep-deprived state is the failing to respond in a time restricted manner to a stimulus [37,38].

Nevertheless, we did not find indications that memory and concentration capacity is affected by nocturnal mobile phone use. One explanation, suggested in a meta-analysis from Pilcher et al.[39], could be that the effects of sleep deprivation have greater influences on feelings of fatigue and other related mood conditions than on cognitive performance. Other explanations could be that these two cognitive tests are not sensitive enough or the sample size was too small. It could also be that a kind of selection bias is present, meaning that adolescents with a high memory and concentration capacity prefer to use mobile phones at night.

Interestingly, the results of the cognitive concentration test and the self-reported lack of concentration are fairly consistent. Self-reported lack of concentration was the symptom that increased the least when being awakened at least once a month with self-reported and objective recorded mobile phone use during night. However, the correlations between self-reported lack of concentration and cognitive test outcomes were small.

One of the strengths of our study is the consideration of both self-reported and objective operator recorded data on mobile phone use during night. Both have their merits and limitations. Operator recorded data are not subject to reporting bias compared to self-reported data, but there are several issues that have to be considered when using operator recorded data. Received text messages and calls during night were considered in our study. In operator recorded data only calls being answered are recorded, so one can be sure that if an incoming call was recorded, the call was answered. However, we cannot prove that study participants were already asleep when the call was answered, since we did not have information on sleeping times. Concerning text messages, mobile phone operators can only record text messages that are sent through the Short-Message-Service (SMS). Nowadays adolescents mostly connect to WLAN or use the mobile internet connection on their mobile phone to send messages through internet-based apps e.g. “WhatsApp” instead of using the Short-Message-Service. Text messages using internet-based apps cannot be recorded by mobile phone operators. Thus, somewhat different information is collected with operator recorded data compared to self-reported exposure data and thus correlations between these exposure measures were only moderate in our study. Nevertheless, findings were fairly consistent, which suggests that bias from exposure assessment is unlikely.

We made considerable effort to adjust our analyses for relevant confounding factors. Still, there might be some residual confounding. Strikingly, the symptom risk estimates for models with (adjusted 2) and without (adjusted 1) mobile phone use adjustments were similar for operator recorded exposure data and only a little reduced for self-reported exposure data. This suggests that indeed mobile phone induced sleep disturbances play an important role for the observed associations. And other unmeasured factors related to mobile phone use were not found to substantially modify the observed relations between symptoms and mobile phone use during night when taking them indirectly into account in the adjusted 2 model addressing confounding by indication.

Van den Bulck et al.[2] reported that 27% of 13-year-old adolescents were being awakened by mobile phone at least once per month, which was found to be similar in our study (22% of the study participants). They found that the use of mobile phone during night increased the odds of being tired by 3.3 (95% CI:1.8–6.0) in the follow-up investigation [3]. This result was also similar to the OR we found for study participants reporting being responsive during night (OR: 3.04, 95% CI:1.48–6.25). Punamaki et al.[40] found that intensive mobile phone usage in girls was associated with poor perceived health. They propose the same mediating links as we do.

## Conclusion

Among Swiss adolescents, we have observed that nocturnal mobile phone use was associated with an increase in health symptom reports such as tiredness, rapid exhaustibility, headache and physical ill-being, but not with memory and concentration capacity. More studies to investigate these associations are necessary and education in sleep behaviour may be inevitable since the mobile phone is now the most familiar lifestyle factor for adolescents.

Public Health prevention strategies should focus on helping adolescents set limits for their accessibility by mobile phone, especially during night.
